# Supplementary material for: Disruptions to youth contraceptive use during COVID-19: Mixed-methods results from Nairobi, Kenya
Source: PLOS Glob Public Health. 2023 Feb 23;3(2):e0001005. doi: 10.1371/journal.pgph.0001005 (PMC10021138; doi:10.1371/journal.pgph.0001005)
Supplement: Attachment — Submitted filename: COVIDImpactFP_ResponsetoReviewers_09.30.22.docx [file pgph.0001005.s001.docx]

Dear Dr. Malca and PLOS Global Health Reviewers,

Thank you for your positive review of our manuscript entitled “Disruptions to youth contraceptive use during COVID-19: mixed-methods results from Nairobi, Kenya”. We are pleased to submit a revision that addresses the thoughtful reviewer and editorial feedback. Please see point-by-point responses below.

1. **Describe if you have done the pr-test. If so, include the information.**

**Authors’ Response:** Many thanks for this question, however, we are unclear what the Editor and Reviewer mean by “pr-test.” We clarify that we have completed probability testing for each model and included p-values therein. We are happy to provide additional feedback pending clarification.

2. **Describe how was the sampling for the qualitative component**

**Authors’ Response:** Thank you for this question. We clarify that youth participants and youth-serving stakeholders were identified by community-partnered recruitment through the assistance of local youth organizations; participation in the baseline survey was not a requirement for youth to be selected for the qualitative study (pg. 6, lines 7-9).

3. **How was the verbal consent documented (for example, was recorded?)**

**Authors’ Response:** We clarify that for the quantitative phone surveys, the consent form was discussed with the participant by the residential enumerator, followed by a verbal inquiry, “Do you consent to participate in today’s survey?”, the results of which were recorded by the resident enumerator in the ODK form on her phone or tablet. For the focus group discussions, the qualitative research assistant called each participant at an arranged time three days prior to the focus group discussion. At this time, the verbal consent was conducted and documented on a participant call log. During the Zoom session for the focus group discussion, the moderator reminded the group that each had verbally consented individually and asked participants if they changed their mind regarding participation. We have added details regarding oral consent documentation on pg. 6, lines 30-31.

4. **How was decided the compensation for the study participants.**

**Authors’ Response:** Compensation was decided during the proposal development by the JHSPH and ICRHK teams following discussions with community partners and PMA resident enumerators (pg. 6, lines 32-33). Through consultation, the researchers settled on an amount that was deemed reasonable and not too high to be considered as undue enticement. Compensation amounts were comparable to amounts the team had provided in other studies. The amount was documented on the consent forms and submitted for approval by ethics review committee.

5. **How do you assure confidentiality and privacy using zoom video? Cameras were on?**

**Authors’ Response:** Extensive protections were in place for privacy and confidentiality for the zoom-based discussions, following best practices (pg. 6, lines 35-36). Specifically, for qualitative study activities that used Zoom, the Zoom meeting call-in information, including the meeting phone number, meeting web address, brief information on how to access to password-protected Zoom meeting, and an individual non-identifiable ID (i.e. Desktop1), were sent immediately before the start of the study activity in order to prevent other people from learning of the meeting phone number/link and trying to call in. Participants were provided with instructions on how to log into the meeting and how to change their display name to their non-identifiable ID. Moderators/interviewers and participants were be instructed to use the most updated version of Zoom, prior to any meetings.

FGD moderators and IDI/KII interviewers established the following default settings when scheduling Zoom meetings to ensure that upon entry:

- Participants were required to enter a password unique to that meeting
- Participants were placed in a meeting “waiting room” before they could enter the FGD or interview, and
- Participants’ video was turned off.

Moderators and interviewers also disabled the following advanced settings in Zoom before granting participants entry to the FGD/interview: participants’ ability to share their screen, ability to chat with other participants using the chat function, and ability to change their display name.

The Research Assistant asked a series of questions to confirm privacy for the participants. The RA also asked the participants to designate a “code word” to share in the event that another person came into hearing range of the discussion.

6. **How did you analyze independent variables with list of items like wealth; this is not clearly stated in the text.**

**Authors’ Response:** We clarify that each independent variable (sociodemographic, contraceptive, and COVID-19-related correlates) was analyzed separately prior to inclusion in multivariable models, as indicated on pg. 5, lines 32-36: “Unadjusted and adjusted logistic regression models, using GLM link log and family binomial, examined sociodemographic, contraceptive, and COVID-19-related correlates of contraceptive disruption overall and by gender; adjusted models included correlates with p<0.1 from bivariate models (overall or gender stratified), after assessing for multicollinearity.”

7. **Waived parental consent was used, so if the respondent with no parent, what would be done**

**Authors’ Response:** Once the approval for waiver was given by Ethical Review Committee, only minors were only required to give informed assent to participate in the study. Therefore, even for respondents with no parents, only the minor consent was required.

8. **Add reflection regarding the limitations of inability to compare to pre-COVID contraceptive difficulties or potentially post pandemic.**

**Authors’ Response:** We have elaborated on our limitation surrounding inability to compare to pre-COVID contraceptive difficulties and urged for continuous monitoring in the post-COVID periods (pg. 12, lines 3-5).

**Journal Requirements:**

1. **Please provide a/amend your detailed Financial Disclosure statement. This is published with the article. It must therefore be completed in full sentences and contain the exact wording you wish to be published.**
a. **Please clarify all sources of funding (financial or material support) for your study. List the grants (with grant number) or organizations (with url) that supported your study, including funding received from your institution.**
b. **State the initials, alongside each funding source, of each author to receive each grant.**
c. **State what role the funders took in the study. If the funders had no role in your study, please state: “The funders had no role in study design, data collection and analysis, decision to publish, or preparation of the manuscript.”**
d. **If any authors received a salary from any of your funders, please state which authors and which funders.**

**Authors’ Response:** We have added these details immediately following the references.

2. **In the online submission form, you indicated that "Quantitative data are available upon request from pmadata.org. To maximize participant privacy, qualitative data are not available.". All PLOS journals now require all data underlying the findings described in their manuscript to be freely available to other researchers, either 1. In a public repository, 2. Within the manuscript itself, or 3. Uploaded as supplementary information.**

**This policy applies to all data except where public deposition would breach compliance with the protocol approved by your research ethics board. If your data cannot be made publicly available for ethical or legal reasons (e.g., public availability would compromise patient privacy), please explain your reasons by return email and your exemption request will be escalated to the editor for approval. Your exemption request will be handled independently and will not hold up the peer review process, but will need to be resolved should your manuscript be accepted for publication. One of the Editorial team will then be in touch if there are any issues.**

**Authors’ Response:** We clarify that public deposition of qualitative data would breach compliance with the protocol approved by our research ethics board, as it would compromise participant privacy. We are seeking an exemption request from the Editorial team.

3. **Please provide separate figure files in .tif or .eps format. Please remove the embedded figure from the manuscript file.**

**Authors’ Response:** We have provided the figure files in .tif and removed the embedded figures from the manuscript file.

4. **We have noticed that you have uploaded Supporting Information files, but you have not included a list of legends. Please add a full list of legends for your Supporting Information files after the references list.**

**Authors’ Response:** We clarify that the Supporting Information files are provided for review only and are not for publication, per our previous correspondence with the Editorial Staff.

5. **Please review your reference list to ensure that it is complete and correct. If you have cited papers that have been retracted, please include the rationale for doing so in the manuscript text, or remove these references and replace them with relevant current references. Any changes to the reference list should be mentioned in the rebuttal letter that accompanies your revised manuscript. If you need to cite a retracted article, indicate the article’s retracted status in the References list and also include a citation and full reference for the retraction notice.**

**Authors’ Response:** We have reviewed the reference list to ensure it is complete and correct.
