# Supplementary material for: Disruptions to youth contraceptive use during COVID-19: Mixed-methods results from Nairobi, Kenya
Source: PLOS Glob Public Health. 2023 Feb 23;3(2):e0001005. doi: 10.1371/journal.pgph.0001005 (PMC10021138; doi:10.1371/journal.pgph.0001005)
Supplement: Attachment — Submitted filename: COVIDImpactFP_ResponsetoReviewers_12.01.22.docx [file pgph.0001005.s002.docx]

Dear Dr. Malca and PLOS Global Health Reviewers,

Thank you for your positive review of our manuscript entitled “Disruptions to youth contraceptive use during COVID-19: mixed-methods results from Nairobi, Kenya”. We are pleased to submit a revision that addresses the thoughtful reviewer and editorial feedback. Please see point-by-point responses below.

1. **Please review your reference list to ensure that it is complete and correct. If you have cited papers that have been retracted, please include the rationale for doing so in the manuscript text, or remove these references and replace them with relevant current references. Any changes to the reference list should be mentioned in the rebuttal letter that accompanies your revised manuscript. If you need to cite a retracted article, indicate the article’s retracted status in the References list and also include a citation and full reference for the retraction notice.**

**Authors’ Response:** We confirm that our reference list is complete and correct. To our knowledge, we have not cited any retracted papers.

2. **Authors must clarify some procedures, considering ethical principles (confidentiality, not harm, autonomy).**

**Authors’ Response:** Thank you for this opportunity to clarify our ethical procedures. We clarify that all procedures were in line with ethical best practices for sensitive topics, including specialized training to ensure confidentiality, privacy protections (auditory privacy screener screener and protocol), and provision of resource referrals (COVID-19 and violence support) (pg. 7, lines 10-13). We have further responded to specific points regarding privacy below.

3. **Clarify if they have performed a pre-test; if so include it**

**Authors’ Response:** We clarify that the original questionnaire was pre-tested for initial cohort data collection, however, given the timing of the 2020 follow-up survey during the COVID-19 pandemic, pre-testing with additional participants was not undertaken. Given that the focus of this manuscript is on 2020 data collection and no pre-test was performed, these details are not included within the manuscript.

4. **Clarify the criteria to include your sample for qualitative part**

**Authors’ Response:** Thank you for the opportunity to clarify the criteria for the qualitative sample. Eligibility criteria were as follows: 1) youth FGDs—15-24 years old, unmarried, living in Nairobi county for at least one year; 2) youth-serving stakeholder FGDs—currently or recently working at a youth-serving organization in a role in direct contact with youth, in Nairobi county, or otherwise knowledgeable about youth health issues in Nairobi; 3) key informant interviews—health and social service officials and other government officers managing or providing services in the area of adolescent/youth health, social supports, gender equity, and microfinance/economic empowerment; and 4) youth IDIs—youth participant in YRDSS cohort with completion of 2020 quantitative survey (pg. 6, lines 12-19).

5. **Explain how do you assure confidentiality and privacy using zoom video as means of data collection method**

**Authors’ Response:** We clarify that Zoom video was never used, only Zoom audio. We outline further points below and have included an abbreviated version of these details in the manuscript (pg. 6, lines 22-26).

For qualitative study activities that used Zoom, the Zoom meeting call-in information, including the meeting phone number, meeting web address, brief information on how to access to password-protected Zoom meeting, and an individual non-identifiable ID (i.e. Desktop1), were sent immediately before the start of the study activity in order to prevent other people from learning of the meeting phone number/link and trying to call in. Participants were provided with instructions on how to log into the meeting and how to change their display name to their non-identifiable ID. Moderators/interviewers and participants were be instructed to use the most updated version of Zoom, prior to any meetings.

FGD moderators and IDI/KII interviewers established the following default settings when scheduling Zoom meetings to ensure that upon entry:

- Participants were required to enter a password unique to that meeting
- Participants were placed in a meeting “waiting room” before they could enter the FGD or interview, and
- Participants’ video was turned off.

Moderators and interviewers also disabled the following advanced settings in Zoom before granting participants entry to the FGD/interview: participants’ ability to share their screen, ability to chat with other participants using the chat function, and ability to change their display name.

The Research Assistant asked a series of questions to confirm privacy for the participants. The RA also asked the participants to designate a “code word” to share in the event that another person came into hearing range of the discussion.

6. **How did you analyze independent variables with list of items like wealth; not clearly stated**

**Authors’ Response:** We clarify that our wealth variable did not use a household index, such as in the Demographic and Health Surveys; rather, this was a self-reported household wealth ladder, on a scale from 0-10, and was then analyzed as tertiles. We have added these details, as well as analysis categories for the other demographic variables (pg. 5, lines 12-17).

7. **Waived parental consent was used so if respondent with no parent what would be done?**

**Authors’ Response:** We clarify that parental consent was waived. Therefore, once the approval for waiver was given by Ethical Review Committee, only minors were only required to give informed assent to participate in the study—no parental consent was required. Therefore, even for respondents with no parents, only the minor consent was required. Participant only consent has been clarified in text (pg. 7, line 8).

8. **Make recommendation specific to your finding you were not study out come of contraceptive disruption like the risk of unintended pregnancy, unsafe abortion, further aspiration to use contraception.**

**Authors’ Response:** Thank you for this point, and we hope that we are correctly interpreting it. We clarify that we have edited the final sentence of the Discussion to ensure that the recommendation is specific to our findings. Specifically, we call for continued data monitoring on contraceptive disruptions; such data will be necessary to better understand impact on unintended pregnancy and long-term aspirations (pg. 13, lines 4-6). We are happy to clarify further if we have misinterpreted the Editor’s recommendation.
